# GLUT-1 may predict metastases and death in patients with locally advanced rectal cancer

**DOI:** 10.3389/fonc.2023.1094480

**Published:** 2023-03-09

**Authors:** Tae Hyun Kim, Yoonjin Kwak, Changhoon Song, Hye Seung Lee, Duck-Woo Kim, Heung-Kwon Oh, Jin Won Kim, Keun-Wook Lee, Sung-Bum Kang, Jae-Sung Kim

**Affiliations:** ^1^ Department of Radiation Oncology, Seoul National University College of Medicine, Seoul, Republic of Korea; ^2^ Department of Pathology, Seoul National University College of Medicine, Seoul, Republic of Korea; ^3^ Department of Radiation Oncology, Seoul National University College of Medicine, Seoul National University Bundang Hospital, Seongnam, Republic of Korea; ^4^ Department of Surgery, Seoul National University College of Medicine, Seoul National University Bundang Hospital, Seongnam, Republic of Korea; ^5^ Department of Internal Medicine, Seoul National University College of Medicine, Seoul National University Bundang Hospital, Seongnam, Republic of Korea

**Keywords:** Glucose transporter-1 (GLUT-1), neoadjuvant chemoradiation (CRT), rectal cancer (RC), survival, metastasis

## Abstract

**Introduction:**

Glucose transporter-1 (GLUT-1) has been studied as a possible predictor for survival outcomes in locally advanced rectal cancer (LARC).

**Methods:**

We aimed to investigate the prognostic role of GLUT-1 in LARC using the data of 208 patients with clinical T3–4 stage and/or node-positive rectal adenocarcinoma, all of whom underwent neoadjuvant chemoradiotherapy (CRT) and subsequent total mesorectal excision (TME). Both pre-CRT and post-CRT specimens were immunohistologically stained for GLUT-1. Patients were classified into GLUT-1-positive and GLUT-1-negative groups and distant metastasis-free survival (DMFS) and overall survival (OS) was analyzed and compared.

**Results:**

At a median follow-up of 74 months, post-CRT GLUT-1 status showed a significant correlation with worse DMFS (p=0.027, HR 2.26) and OS (p=0.030, HR 2.30). When patients were classified into 4 groups according to yp stage II/III status and post-CRT GLUT-1 positivity [yp stage II & GLUT-1 (-), yp stage II & GLUT-1 (+), yp stage III & GLUT-1 (-), yp stage III & GLUT-1 (+)], the 5-year DMFS rates were 92.3%, 63.9%, 65.4%, and 46.5%, respectively (p=0.013). GLUT-1 (-) groups showed markedly better outcomes for both yp stage II and III patients compared to GLUT-1 (+) groups. A similar tendency was observed for OS.

**Discussion:**

In conclusion, post-CRT GLUT-1 may serve as a prognostic marker in LARC.

## Introduction

The hypoxic tumor microenvironment induces various reactions in tumor cells, including increased glucose utilization, resulting in increased anaerobic glycolysis (1). Consequently, glucose transporters (GLUTs) also exhibit high levels of expression, with GLUT-1 to GLUT-4 playing the most important role in glucose regulation (2). GLUTs have specific locations according to their subtypes, and certain types are only expressed in certain cell lines. GLUT-1, for instance, is mainly expressed in endothelial cells of the blood–brain barrier, but it has also been found to be upregulated in various tumor cells (3–6). Moreover, the expression level of GLUT-1 has been reported to be increased by oncogenes such as Ras and Src and by transcription factors involved in tumorigenesis such as SIX1 (7, 8). In light of these findings, GLUT-1 is currently being studied as a surrogate marker for tumor prognosis. In fact, some prior studies have shown an association between survival outcomes and GLUT-1 in various tumor types, including breast, lung, and pancreatic cancers (9–11).

However, the results of prior studies regarding colorectal cancers are not consistent. GLUT-1 has been shown to be associated with worse disease-specific mortality, overall survival, and tumor regression grade in some studies (12–15); others have reported statistically nonsignificant results (16–19), and one study reported a positive effect of GLUT-1 on overall survival (20). The agreement between studies is further reduced when the scope is narrowed to rectal cancer, where the number of patients per study and the absolute number of studies are both low (13–16, 18, 19). Additionally, for locally advanced rectal cancer (LARC), the majority of the studies only utilized either pre-neoadjuvant chemoradiotherapy (pre-CRT) specimens or post-neoadjuvant chemoradiotherapy (post-CRT) specimens, suggesting the need for a more comprehensive analysis using both sample types.

Based on the pitfalls presented above, this study aimed to investigate the correlation between GLUT-1 and survival outcomes in LARC using pre-CRT and post-CRT samples and to investigate distant metastasis-free survival (DMFS) and overall survival (OS).

## Methods

### Patients

A total of 208 patients from a single institution with histologically confirmed rectal adenocarcinoma, clinical T3–4 and/or node-positive or low-lying T2 were recruited between 2004 and 2012. All patients were newly diagnosed and free of metastasis. Patients received neoadjuvant CRT followed by total mesorectal excision (TME) in 6 to 8 weeks. Radiation therapy was delivered in 5.5 weeks (45 Gy to the pelvis in 25 fractions and 5.4 Gy to the primary lesion in 3 fractions). The chemotherapy regimens were one of the three; 5-fluorouracil with leucovorin (FL), capecitabine, or 5-fluorouracil with leucovorin and oxaliplatin (FOLFOX). TME was performed in one of the three ways: low anterior resection, ultralow anterior resection or abdominoperineal resection. Adjuvant chemotherapy was recommended after resection for all patients who were medically fit. The median follow-up time was 74 months (8-164 months). This study was approved by the Seoul National University Bundang Hospital Institutional Review Board. Written informed consent was waived due to the retrospective design of the study, but the patients were anonymized to protect privacy. The waiver of informed consent was authorized by Seoul National University Bundang Hospital Institutional Review Board (B-2109-707-106). All methods of this study were performed in accordance with the relevant guidelines and regulations.

### Tissue sampling and immunohistochemistry

**Figure 1:**
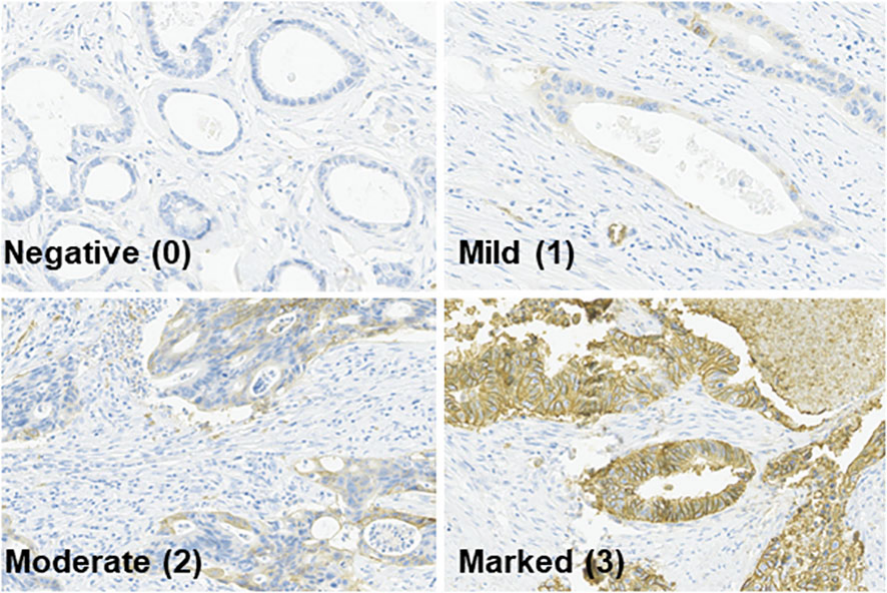
Immunohistochemical staining intensity of GLUT-1 (x200).

Tissue sampling was performed twice: before neoadjuvant CRT using colonoscopic biopsy (pre-CRT) and during surgery using a surgical specimen (post-CRT). The samples were stained for GLUT-1 using immunohistochemistry with an antibody from Abcam (Cambridge, United Kingdom). The histological score (H-score) for each pre-CRT and post-CRT specimen was calculated by multiplying the percentage of GLUT-1 staining in the specimen by the intensity of GLUT-1 staining in the same specimen. The percentage of GLUT-1 staining was scored from 0 to 100, whereas the intensity of GLUT-1 staining was scored from 0 to 3 (0 denoted negative staining and 3 denoted maximum staining, **Figure 1**). The immunohistochemical evaluation was performed by one dedicated GI pathologist (Y.K). GLUT-1 expression was considered positive when the H-score was equal to or greater than 1 and was considered negative when the H-score was less than 1.

### Statistical analysis

The log-rank test was used to compare survival curves between the GLUT-1-positive and GLUT-1-negative groups. Additionally, factors with p value <0.1 in the univariate analysis using Cox proportional hazards model were used to construct a multivariate Cox proportional hazards model and calculate the hazard ratio for each factor. All variables were transformed into categorical variables using conventional or clinically significant cutoffs. The baseline CEA value was classified as low or high using 5 ng/ml as the cutoff value. ypT stage was grouped as 0-2 or 3-4, whereas ypN stage was grouped as 0 or 1-2. Statistical analysis was performed with R software 4.1.0, and statistical significance was indicated by p<0.05.

## Results

### Patient characteristics

The median age of the patients was 60.6 years (range, 49.1-72.1, **Table 1**). The majority of tumors were diagnosed as clinical T3 stage (n=176, 84.6%), and clinical lymph node involvement was seen in most of the patients (n=171, 82.2%) (**Table 1**). Nearly 90% of patients received either low anterior resection or ultralow anterior resection, classified as sphincter-preserving surgery (n=186, 89.4%). Following radical surgery, 178 patients (85.6%) received 5-FU–based adjuvant chemotherapy. In terms of pathologic stage, although half of the patients remained in the ypT3 stage (n=109, 52.4%), approximately a quarter of patients were diagnosed as ypT2 stage (n=57, 27.4%), and some showed a complete response (n=33, 15.9%). Similarly, the majority of the patients were diagnosed as ypN0 regarding ypN stage (n=134, 64.7%). The planned radiation dose was 50.4Gy for all patients, and the planned dose was delivered to all but 1 patient who terminated early. Majority of the patients received chemotherapy in either FL (n=91, 43.7%) or capecitabine (n=107, 51.4%). There was no premature termination of chemotherapy.

**Table 1:** Patient characteristics and clinical/pathologic staging.

Characteristics	Total (n=208)			
	N		%	
Age (years)	60.6 ± 11.5			
Sex				
Female	71		34.1%	
Male	137		65.9%	
cT stage				
2	16		07.7%	
3	176		84.6%	
4	16		07.7%	
cN stage				
Negative	37		17.8%	
Positive	171		82.2%	
Baseline CEA (ng/ml)				
Low (<5)	146		70.2%	
High (≥5)	62		29.8%	
Operation				
APR	22		10.6%	
SPS	186		89.4%	
ypT stage				
0	33		15.9%	
1	6		02.9%	
2	57		27.4%	
3	109		52.4%	
4	3		01.4%	
ypN stage				
0	135		64.9%	
1	53		25.6%	
2	20		09.7%	
Pathologic Grade				
W/D & M/D	194		93.3%	
P/D	14		06.7%	
Planned Radiation dose (Gy)	50.4 ± 0.0			
Applied Radiation dose (Gy)	50.2 ± 3.0			
Concurrent Chemotherapy Regimen				
FL	91		43.7%	
Capecitabine	107		51.4%	
FOLFOX	2		1.0%	
Others	8		3.8%	

CEA, carcinoembryonic antigen; APR, abdominal perineal resection; SPS, sphincter preserving surgery; W/D, well differentiated; M/D, moderately differentiated; P/D, poorly differentiated; FL, 5-fluorouracil and leucovorin; FOLFOX, 5-fluorouracil, leucovorin, and oxaliplatin.

**Figure 2:**
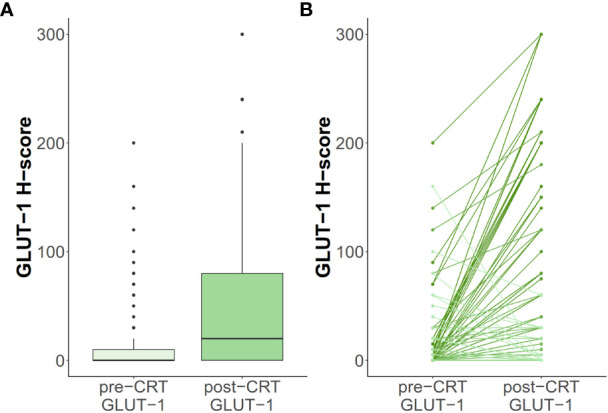
Changes of GLUT-1 H-score are depicted **(A)** in box plot and **(B)** among individual patients.

Regarding GLUT-1 status, the median H-scores for pre-CRT and post-CRT specimens were 0 and 12.5, respectively, indicating an increasing tendency after neoadjuvant therapy (**Figure 2**). The percentages of positive staining were 38% and 61%, in alignment with the increasing tendency of the H-score.

### Correlation between GLUT-1 and survival outcomes

**Figure 3:**
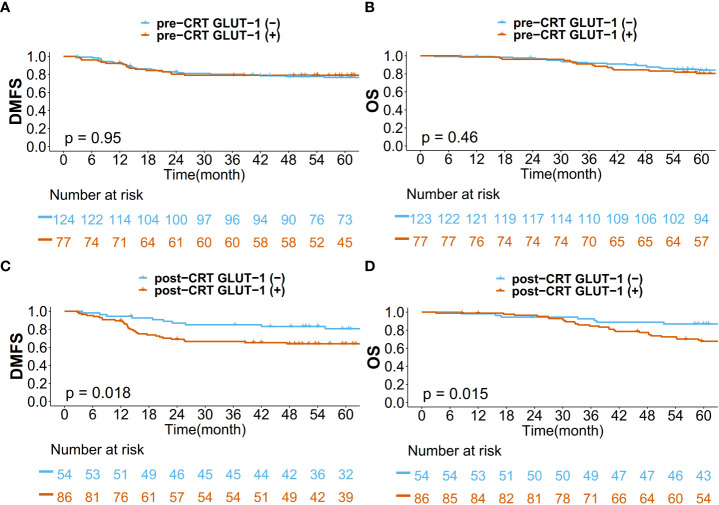
Kaplan–Meier curves for **(A)** DMFS based on pre-CRT status, **(B)** OS based on pre-CRT status, **(C)** DMFS based on post-CRT status, and **(D)** OS based on post-CRT status.

Kaplan–Meier curves for DMFS/OS were generated based on GLUT-1 expression status and treatment phase (pre-CRT/post-CRT) (**Figure 3**). The pre-CRT GLUT-1 status did not result in a statistically significant difference for DMFS and OS. However, there was a significant difference between the post-CRT GLUT-1-positive group and the post-GLUT-1-negative group in terms of both DMFS (p=0.018) and OS (p=0.015). The actuarial 5-year DMFS rate of patients was 63.9% for the post-CRT GLUT-1-positive group and 80.6% for the post-CRT GLUT-1-negative group. For 5-year OS, the value was 66.7% for the GLUT-1-positive group and 86.9% for the GLUT-1-negative group. For both DMFS and OS, positive post-CRT GLUT-1 staining was significantly associated with worse prognosis.

Univariate Cox proportional hazards analysis was performed using clinical information (**Table 2**). Age, sex, baseline CEA, ypT stage, ypN stage, pathologic grade, and post-CRT GLUT-1 status were included. Age, sex, and pathologic grade showed nonsignificant p values in the univariate analysis. Multivariate analysis was conducted with the factors with p value <0.1 in the univariate analysis (**Table 3**). The multivariate analysis revealed that baseline CEA, ypN stage, and post-CRT GLUT-1 status were significant predictors for DMFS, and ypN stage and post-CRT GLUT-1 status were the only predictors that met the statistical criteria for predicting OS. The hazard ratios for post-CRT GLUT-1 status were 2.26 (p=0.027) and 2.30 (p=0.030) for DMFS and OS, respectively; these values were higher than those for baseline CEA for both clinical outcomes.

**Table 2:** Results of univariate Cox analysis for distant metastasis-free survival and overall survival.

Characteristics	Distant metastasis-free survival		Overall survival	
	Hazard ratio (95% CI)	p value	Hazards ratio (95% CI)	p value
Age (year)	1.00 (0.97-1.02)	0.930	1.02 (0.99-1.05)	0.133
Sex				
Female	Reference		Reference	
Male	1.04 (0.57-1.91)	0.894	1.68 (0.85-3.33)	0.135
Baseline CEA (ng/ml)				
Low (<5)	Reference		Reference	
High (≥5)	1.01 (1.00-1.01)	<0.001	2.72 (1.51-4.92)	0.001
ypT stage				
0-2	Reference		Reference	
3-4	5.13 (2.39-10.98)	<0.001	5.82 (2.59-13.08)	<0.001
ypN stage				
0	Reference		Reference	
1-2	4.63 (2.53-8.47)	<0.001	5.23 (2.77-9.88)	<0.001
Pathologic Grade				
W/D & M/D	Reference		Reference	
P/D	1.03 (0.32-3.31)	0.962	1.15 (0.36-3.71)	0.816
Post-CRT GLUT-1 status				
Negative	Reference		Reference	
Positive	2.31 (1.13-4.72)	0.021	2.44 (1.16-5.13)	0.019

CEA, carcinoembryonic antigen; W/D, well differentiated; M/D, moderately differentiated; P/D, poorly differentiated; GLUT-1, glucose transporter-1.

**Table 3:** Results of multivariate Cox analysis for distant metastasis-free survival and overall survival.

Characteristics	Distant metastasis-free survival		Overall survival	
	Hazard ratio (95% CI)	p value	Hazards ratio (95% CI)	p value
Baseline CEA (ng/ml)				
Low (<5)	Reference		Reference	
High (≥5)	1.01 (1.00-1.01)	0.009	1.89 (0.99-3.63)	0.550
ypT stage				
0-2	Reference		Reference	
3-4	2.24 (0.91-5.50)	0.079	2.21 (0.83-5.93)	0.114
ypN stage				
0	Reference		Reference	
1-2	2.51 (1.28-4.90)	0.007	3.02 (1.51-6.06)	0.002
Post-CRT GLUT-1 status				
Negative	Reference		Reference	
Positive	2.26 (1.10-4.65)	0.027	2.30 (1.08-4.89)	0.030

CEA, carcinoembryonic antigen; GLUT-1, glucose transporter-1.

### Subgroup analysis based on yp stage II and III and post-CRT GLUT-1 status

**Figure 4:**
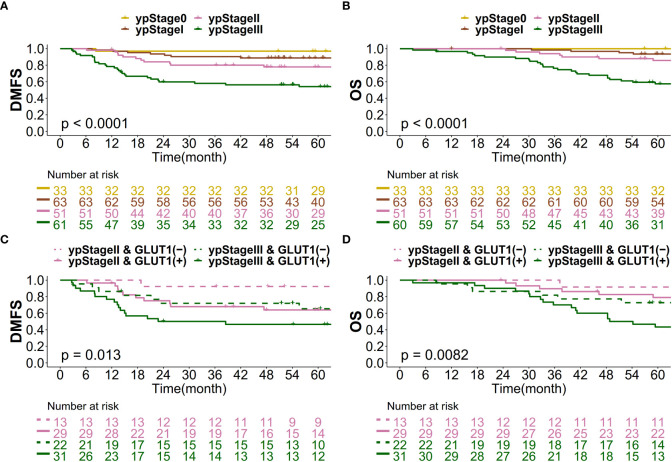
Kaplan–Meier curve for **(A)** DMFS based on yp stage, **(B)** OS based on yp stage, **(C)** DMFS based on the new prognostic grouping, and **(D)** OS based on the new prognostic grouping.

When patients were classified according to yp stage, approximately a quarter accounted for yp stage II (n=51, 24.5%) and another quarter accounted for yp stage III (n=60, 28.8%). The 5-year actuarial DMFS rates for patients in yp stages 0, I, II, and III were 97.0%, 88.7%, 77.9% and 54.1%, respectively (**Figure 4A**). For 5-year actuarial OS, the values were 100%, 93.5%, 85.8% and 57.5%, respectively (**Figure 4B**). For patients in yp stages II and III, patients were classified into 4 groups according to yp stage II/III and post-CRT GLUT-1 status (**Figures 4C, D**): yp stage II & GLUT-1 (-), yp stage II & GLUT-1 (+), yp stage III & GLUT-1 (-), and yp stage III & GLUT-1 (+). For yp stage II patients, yp stage II & GLUT-1 (-) group showed a more favorable 5-year actuarial survival rates of 92.3% and 91.7% for both DMFS and OS whereas the yp stage II & GLUT-1 (+) group showed a less favorable 5-year actuarial survival rates of 63.9% and 79.0%. This tendency was maintained in the yp stage III patients as well; 5-year actuarial DMFS and OS for yp stage III & GLUT-1(-) group was 65.4% and 72.7% when the values were 46.5% and 43.4% for yp stage III & GLUT-1 (+) group. Taken altogether, yp stage II & GLUT-1 (-) group showed the most favorable outcomes, whereas the yp stage III & GLUT-1 (+) group showed the least favorable 5-year actuarial survival rates. The yp stage II & GLUT-1 (+) and yp stage III & GLUT-1 (-) group together showed intermediate outcomes. The new prognostic grouping based on post-CRT GLUT-1 status was shown to be statistically significant for predicting both distant metastasis and overall survival (p=0.0130 and p=0.0082).

## Discussion

The findings of our study indicate that post-CRT GLUT-1 status is significantly associated with DMFS and OS in patients with LARC. Furthermore, post-CRT GLUT-1 status has shown the possibility of serving as a prognostic subgroup together with ypN stage.

Of particular interest is the increase in GLUT-1 H-scores after CRT in our study. One possible hypothesis is that GLUT-1 is mainly located at the central hypoxic region of the tumor, and the peripheral normoxic region with low GLUT-1 levels was eradicated during the treatment. Considering that H-score was calculated by multiplying the percentage of GLUT-1 staining in the specimen by the intensity of GLUT-1 staining in the same specimen, the increase in percentage of GLUT-1 staining due to eradication of peripheral normoxic region may have resulted in higher H-score in the post-CRT specimen, although it is not possible to confirm such phenomenon directly from current study. The central distribution of GLUT-1 after CRT in rectal cancer has already been reported in one prior study (21). This hypothesis is further supported by the results of another study that further showed that GLUT-1 immunohistochemistry staining conducted on the superficial part of the tumor had no prognostic capability, whereas GLUT-1 staining in a deep part of the tumor could indicate OS (13).

The main mechanism behind the strong association between GLUT-1 and survival outcomes is thought to be tumor hypoxia. Various studies have suggested worse clinical outcomes in hypoxic tumors using markers such as HIF1-a, CA-9 and hemoglobin levels in LARC (16, 19, 22). Since tumor hypoxia plays a key role in both early and late tumor metastasis by promoting invasion, migration, angiogenesis, and adaptation to the metastasis site, it is likely to decrease OS by promoting distant metastasis (23). This is in alignment with the fact that distant metastasis is the primary cause of treatment failure in LARC patients and with the findings of a prior study where GLUT-1 was reported to be associated with distant metastasis but not regional recurrence (13, 24).

Regarding the prognostic subgroup, the large difference in survival in patients with the same yp stage is worth noting. Such a phenomenon has also been observed in our prior study, where yp stage II and III rectal cancer patients were classified into good response (GR) and poor response (PR) groups according to the Dworak tumor regression grade (25). In the abovementioned study, the yp stage II & GR group showed similar survival results as the yp stage 0-I group, while the yp stage III & GR group showed similar survival results as the yp stage II group. This, together with the subgroup analysis of our study, implies the heterogeneity of yp stage II and III rectal cancer patients and thus calls for a more customized treatment according to each patient’s prognosis.

Meanwhile, circulating tumor DNA (ctDNA) is an emerging biomarker that has gained popularity nowadays. ctDNA can be a prognostic and predictive biomarker in gastrointestinal cancers (26–28). Recently, Kotani et al. have reported results from GALAXY, which is an observational arm of the ongoing CIRCULATE-Japan study (UMIN000039205) that analyzed preoperative and postoperative ctDNA in patients with stage II-IV resectable colorectal cancer (28). In the multivariate analysis for recurrence in patients with pathologic stage II-III, ctDNA positivity (at 4 weeks after surgery) was the most significant prognostic factor for recurrence (HR 10.82, 95% CI 7.07-16.6, p<0.001). All other clinicopathologic factors including pathologic N stage, MSI, BRAF, and RAS status were not significant. Furthermore, postsurgical ctDNA positivity was predictive of response to adjuvant chemotherapy (HR 6.59, 95% CI 3.53–12.3, p<0.0001). A collaborative study from Memorial Sloan Kettering Cancer Center and University of South Florida have reported that monitoring ctDNA may lead to a faster response assessment compared with traditional radiologic assessment in patients with anal cancer after definitive chemoradiotherapy (29). The median time to molecular ctDNA remission (30 days) was significantly shorter than the median time to complete clinical response (136 days). However, in rectal cancer, it is difficult to conclude as to the prognostic value of ctDNA as most studies are small, the statistical assumptions are dubious and the follow-up period is rather short in some of the included studies. In our current study, we showed the added prognostic value of post-CRT GLUT-1 by immunohistochemistry over conventional staging system. This method is affordable and can be applied to the real clinical practice right away.

Our study has some limitations. First, there is a possibility of inappropriate representation of GLUT-1 status in pre-CRT specimens derived from biopsy. This is a limitation that has been mentioned in a prior study (15). To overcome this issue, the lowest possible cutoff margin was used to avoid precluding any sign of positive GLUT-1 status. Second, this is a retrospective study conducted in a single institution. Although the data structure was designed to make up for the shortcomings of prior studies, a prospective trial with a larger number of patients is required for a more definitive conclusion.

Nonetheless, the results of this study may aid in the utilization of GLUT-1 as a prognostic marker in various ways. Our classification of favorable, intermediate and unfavorable groups according to yp stage II, III and post-CRT GLUT-1 status may serve as a possible grouping system for future trials to determine the optimal chemotherapy regimen in these patients. In addition, we suggest that a more prudent approach might be needed for patients who receive short course radiation therapy and show high levels of GLUT-1 simultaneously. One prior study suggested the possibility of a reduced tumor cell killing in tumor regions with hypoxia when delivering radiation therapy with a hypofractionated schedule (30). Based on this possibility, extra measures such as a higher chemotherapy dose or a more potent chemotherapy regimen might be necessary for patients who receive a short course of radiation therapy but show a high level of GLUT-1.

The utility of GLUT-1 can further be expanded to disease treatment with novel GLUT-1 targeting agents. Combining drugs such as resveratrol or STF-31 that specifically bind to GLUT-1 and conventional cytotoxic agents might facilitate better treatment outcomes in high-risk patients whose tumors exhibit high levels of hypoxia (31).

## Data availability statement

The raw data supporting the conclusions of this article will be made available by the authors, without undue reservation.

## Author contributions

YK and HL provided the data. CS, J-SK and YK were involved in the study design and obtained the raw data. TK and CS conducted statistical analyses. TK & YK drafted the paper, and JS-K and CS reviewed and revised the manuscript. All authors read and approved the final manuscript. CS and JS-K designed the study. All authors contributed to the article and approved the submitted version.
